# The Potential Role of MicroRNAs as Cardioprotective Agents During Cardioplegia: A Preliminary Qualitative Systematic Review

**DOI:** 10.1002/hsr2.72841

**Published:** 2026-07-25

**Authors:** Maryam Baniani, Ali Dabbagh, Pourya Shokri, Firoozeh Madadi

**Affiliations:** ^1^ Shahid Beheshti University of Medical Sciences School of Medicine Tehran Iran; ^2^ Anesthesiology Research Center Shahid Beheshti University of Medical Sciences Tehran Iran

**Keywords:** cardioplegia, cardio‐protection, micro RNA, smart temporal RNA

## Abstract

**Background and Aims:**

Recent advancements in gene expression studies have shed light on the role of non‐coding RNAs in negatively regulating mRNA and inhibiting mRNA translation. Given the significant impact of miRNAs on cardiovascular function, our review aims to comprehensively explore their contribution to mitigating heart injury and their protective effects during cardioplegic‐induced cardiac arrest.

**Methods:**

A systematic search conducted in December 2023 across PubMed, Web of Science, Scopus, and Google Scholar yielded valuable insights.

**Results:**

Our findings indicate that cardioplegia leads to the upregulation of several miRNAs, including miR‐208b, miR‐499‐5p, miR‐10b, miR‐96, miR‐339‐5p, and miR‐483‐3p, alongside the downregulation of miR‐139‐5p, and miR‐194‐5p. Many of these miRNAs are implicated in cardiac pathophysiology, particularly in myocardial injury following cardioplegia in on‐pump cardiac surgery.

**Conclusion:**

While current applications of discovered miRNAs primarily focus on early diagnosis and prognosis of cardiac conditions, their therapeutic potential holds promise for revolutionizing the management of various heart diseases in the future. By elucidating the intricate roles of miRNAs in cardiac health and disease, this review underscores the importance of harnessing their therapeutic potential for improved patient outcomes.

## 1 Introduction

Recent studies on gene expression have revealed some non‐coding RNAs that can had negative regulatory effect on mRNAs and inhibit target mRNAs translation [1, 2]. This non‐coding RNAs found in Eukaryotes as human cells and some viruses [3]. They are classified into different groups based on the number of nucleotides they contain; micro RNAs with 21 to 23 nucleotides and long non‐coding RNAs (Lnc RNAs) with more than 200 nt [1, 4]. Discovering of first miRNA backed to1993 from a study that conducted by Lee RC et. al [5]. Non‐coding RNAs are present in cells and control the expression of cell cycle elements, such as cyclins, cyclin‐dependent kinases (CDKs), CDK inhibitors (CKI) and growth factors [6]. In other hand miRNAs are also found as extracellular circulating molecules [7]. Body fluids contain circulating miRNAs that could serve as biomarkers for various diseases [7]. Various studies showed micro RNAs can play important role in cell to cell communication and create some changes in different cells like:small cells, endothelial cells and also cardio‐myocytes, but the exact mechanism of actions is not clear [8]. However; based on some studies reportage there is some suggestion like great importance role of miRNAs in the pathogenesis of DCM [9]. Different miRNAs like miR‐126, miR‐24, miR‐1, miR‐155, miR‐499, and miR‐199a are found to be associated with various types of heart diseases like coronary artery disease and myocardial infarction [9].

Due to the sharp increase in heart disease and the urgent need for surgery in some patients, various methods have been developed, such as the use of cardiologic drugs. In cardiac surgery, cardioplegic solutions are employed to induce controlled cardiac arrest during operations, thereby enhancing the safety of the procedure [10]. Cardioplegia can be either blood‐based or crystalloid‐based, each having its own perceived advantages and disadvantages. While it is widely acknowledged that cardioplegia leads to cardiac arrest, there is ongoing debate regarding which type of cardioplegic solution offers the highest level of myocardial protection during arrest [10]. Additionally, there is growing interest in exploring other contributing factors, such as micro RNAs, that may play a role in cardio‐protection.

Generally based on highly significant various roles of miRNAs in cardiovascular system we designed this review to investigate meticulously all aspect of contribution of miRNAs in decrease of severity of injury to the heart and protective roles of some micro RNAs during heart arrest by cardioplegic drugs [11].

## 2 Methods

### 2.1 Study Design and Search Strategy

A comprehensive search strategy was designed and conducted in June 2025 for this systematic review. Electronic databases, including PubMed, Web of science, Scopus and google scholar were searched using a combination of following keywords and Medical Subject Headings (MeSH) terms: “micro RNAs,” “small temporal RNAs,” “protection,” “cardioplegia,”. The search strategy was designed to capture studies investigating the role of miRNAs in protection of heart during cardioplegia. References of relevant studies were also reviewed to identify additional eligible studies.

### 2.2 Inclusion and Exclusion Criteria

**Figure 1:**
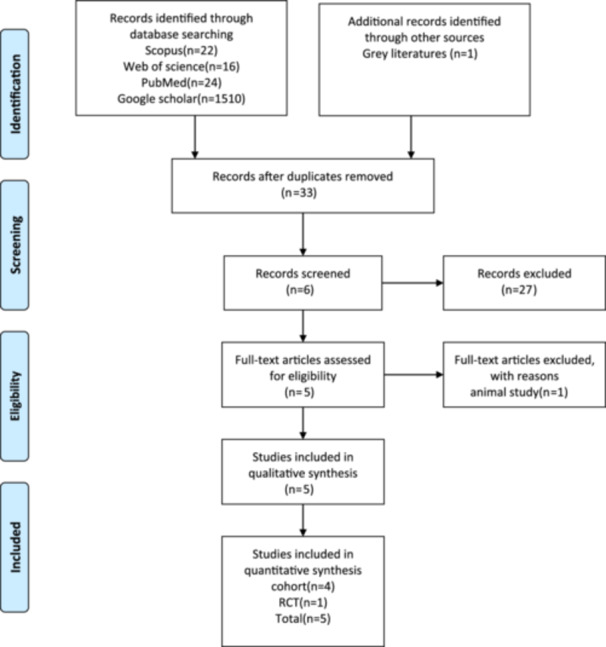
Flowchart based on the PRISMA 2020 guidelines for systematic reviews.

Studies were considered eligible for inclusion if they met the following criteria: (1) original research articles reporting on micro RNAs cardio‐protective roles during cardioplegia, (2) studies providing sufficient data for the analysis, and (3) studies published in peer‐reviewed journals. Case reports, editorials, commentaries, and conference abstracts were excluded (Figure 1).

### 2.3 Study Selection and Data Extraction

Two independent reviewers screened the titles and abstracts of the identified articles to assess their eligibility for inclusion. Any discrepancies were resolved through discussion and consensus. Full‐text articles of potentially relevant studies were retrieved and further assessed for eligibility. Data were extracted from the selected studies using a standardized data extraction form. The following information was collected: study characteristics (e.g., author, year of publication, study design), patient demographics, sample size, type of surgery, miRNA down regulated, miRNA up regulated, and protective effect.

### 2.4 Quality Assessment

Cohort studies were assessed using the Newcastle‐Ottawa Scale (NOS), Clinical trials were evaluated using the Jadad Score.

### 2.5 Statistical Analysis

This study is a qualitative systematic review and does not include primary statistical analyses. Therefore, no statistical tests, significance levels, or software packages were applied or reported. Data synthesis was performed narratively in accordance with qualitative review standards.

## 3 Results

In this study, we conducted a comprehensive investigation of previous studies pertaining to microRNAs and their roles in heart protection during cardioplegia. Ultimately, we identified four cohort studies and one randomized controlled trial (RCT) that addressed this issue with good quality (Tables 1 and 2). The total number of patients included in these studies was 536 that 129 out of them had cardioplegia experiences. Out of the 129 patients included in the study, 29 underwent CABG surgery, 9 underwent aortic valvular surgery, 21 underwent aortic dissection with DHCA, and 70 patients underwent non‐coronary cardiac surgery (Figure 1).

**Table 1:** Risk of bias assessment for the included cohort studies.

	Year 2018	1. Representativeness of the exposed cohort a) truly representative of the average _______________ (describe) in the community ¯b) somewhat representative of the average ______________ in the community ¯c) selected group of users eg nurses, volunteersd) no description of the derivation of the cohort	2. Selection of the non exposed cohort a) drawn from the same community as the exposed cohort ¯b) drawn from a different sourcec) no description of the derivation of the non exposed cohort	3. Ascertainment of exposure a) secure record (eg surgical records) ¯b) structured interview ¯c) written self reportd) no description	4. Demonstration that outcome of interest was not present at start of study a) yes ¯b) no	5. Comparability of cohorts on the basis of the design or analysis a) study controls for _____________ (select the most important factor) ¯b) study controls for any additional factor ¯ (This criteria could be modified to indicate specificcontrol for a second important factor)	6. Assessment of outcome a) independent blind assessment ¯b) record linkage ¯c) self reportd) no description	7. Was follow‐up long enough for outcomes to occur a) yes (select an adequate follow up period for outcome of interest) ¯b) no	8. Adequacy of follow up of cohorts a) complete follow up ‐ all subjects accounted for ¯b) subjects lost to follow up unlikely to introduce bias ‐ small number lost ‐ > ____ % (select anadequate %) follow up, or description provided of those lost) ¯¯c) follow up rate < ____% (select an adequate %) and no description of those lostd) no statement	Overall Score
Xiaohua Wang[12]	2018	c	c	a	a	a	a	a	a	6
Louis A. Saddic[13]	2015	c	c	a	b	b	b	b	d	4
Nobuhiri Mukai[14]	2018	c	c	a	a	a	b	a	a	7
Olof Gidlöf[15]	2013	c	a	a	b	b	a	a	b	6

**Table 2:** Jadad Scale for the included clinical trial.

	1. Was the studydescribed asrandomized (thisincludes the use ofwords such asrandomly, random, andrandomization)?= 1 point^1^	2. Was the studydescribed asdouble blind? = 1 point^2^	3. Was there adescription ofwithdrawals anddropouts?= 1 point	TOTAL JADAD SCORE
Zhou et al. [16]	2	2	1	5

A study by Olof Gidlöf et al. in 2013, involving 411 patients in Sweden, found that miRNAs were elevated in individuals with myocardial infarction (MI) and showed a correlation with left ventricular ejection fraction (LVEF) (*p* < 0.001) [15]. While the ability of miR‐208b (AUC = 0.82) and miR‐499‐5p (AUC = 0.79) to differentiate MI was notable, it was notably lower than that of Troponin T (AUC = 0.95) [15]. Elevated levels of miRNAs were strongly linked to a higher risk of mortality or heart failure within 30 days, with miR‐208b (OR 1.79, 95% CI = 1.38‐2.23, *p* < 0.001) and miR‐499‐5p (OR 1.70, 95% CI = 1.31‐2.20, *p* < 0.001), although this association was no longer significant after adjusting for Troponin T levels [15]. During surgery, miR‐208b and miR‐499‐5p were notably released into the coronary sinus after cardioplegia‐reperfusion at significantly higher levels than in peripheral veins [15].

In another study conducted in Japan, involving 25 patients, significant changes were observed in the expression of several microRNAs in circulating platelets before and after cardiopulmonary bypass [14]. The study found an overexpression of miR‐10b and miR‐96 (miR10b: CI: 1.35 (0.32–2.85), p: 0.01; miR96‐5p: CI: 1.59 (1.06–2.13), *p* < 0.001), which correlated with decreased messenger RNA and protein levels of glycoprotein 1b and vesicle‐associated membrane protein 8 [14]. These changes potentially contribute to platelet dysfunction following cardiopulmonary bypass [14].

In a study involving 9 patients who underwent aortic valvular surgery in the USA, researchers found among,1237identified miRNAs, 21 showed differential expression between baseline and post ischemic left ventricular (LV) samples [13]. The upregulated miRNAs included miR‐339‐5p and miR‐483‐3p, while the downregulated miRNA was miR‐139‐5p (miR‐339‐5p: *p* = 0.016; baseline: 0.88, post ischemia: 2.32, fold change: 2.62; miR‐483‐3p: *p* = 0.016; baseline: 1.14, post ischemia: 4.81, fold change: 4.23; miR‐139‐5p: baseline: 5.80, post ischemia: 3.69) [13].

A study conducted by Wang et al. demonstrated that the values of miR‐194‐5p at 12 h postoperatively were significantly lower than the preoperative values (0.6699 ± 0.3896 vs. 1.2629 ± 0.3622, respectively; *p* = 0.005) [12]. Blood levels of miR‐194‐5p at 12 h postoperatively were almost half of the preoperative levels and remained low until 36 h postoperatively (*p* < 0.0001) [12]. Furthermore, compared to 18 and 24 h postoperatively, the expression of miR‐194‐5p was also significantly lower at 12 h postoperatively (*p* < 0.0001) [12].

A clinical trial conducted in China with 70 patients demonstrated that the protective effect of simvastatin pretreatment on the myocardium may be attributed, at least in part, to the suppression of miR‐15a‐5p expression, leading to an increase in Bcl‐2 mRNA and protein expression [16]. Specifically, simvastatin treatment significantly decreased miR‐15a‐5p expression, as confirmed by quantitative real‐time polymerase chain reaction (qRT‐PCR) analysis (preoperatively: *p* < 0.001; postoperatively: *p* < 0.01) [16].

## 4 Discussion

In this systematic review, we explored the role of microRNAs (miRNAs) in myocardial protection during cardioplegia. While miRNAs have been extensively studied in the context of cardiac pathophysiology, their specific contributions during cardioplegia remain a relatively under‐researched area. Cardioplegia, an essential aspect of cardiac surgery, is associated with ischemia‐reperfusion injury, and miRNAs appear to play a critical role in mitigating the severity of such injuries. Our findings, alongside previous studies, provide important insights into the molecular mechanisms underpinning cardioprotection during and after cardioplegic arrest.

The reviewed studies highlight the dual role of miRNAs as both biomarkers and potential mediators of myocardial protection. In the cardiovascular system, miRNAs control functions of cardiomyocytes, endothelial cells, smooth muscle cells, and fibroblasts. They are key to understanding disorders such as myocardial infarction, hypertrophy, heart failure, arrhythmia, and atherosclerosis, and can serve as therapeutic targets due to their differential expression in diseased tissues and circulation [17].

Certain miRNAs, such as hsa‐miRNA‐1‐3p and miR‐222, are implicated in specific cardiovascular pathologies. For instance, miR‐222 plays a dual role: promoting physiological cardiac growth while reducing pathological hypertrophy and adverse remodeling, making it a potential therapeutic target [18].

Microarray and PCR‐based studies have identified specific microRNAs that are upregulated in response to environmental stress. Some of these microRNAs play protective roles by suppressing pro‐apoptotic signaling, reducing muscle atrophy, and limiting protein translation, while others promote cell cycle arrest and activate MAPK signaling pathways. Interestingly, many of these stress‐responsive microRNAs are also deregulated in various diseases. This connection suggests that leveraging their role in hypometabolism could offer innovative therapeutic strategies for conditions such as cardioplegia [19].

For example, upregulated miRNAs such as miR‐208b and miR‐499‐5p were found to be released in higher concentrations after cardioplegia‐reperfusion, underscoring their association with myocardial stress and recovery [15]. Similarly, the downregulation of miRNAs like miR‐194‐5p and miR‐15a‐5p was linked to cardioprotective effects, particularly in reducing apoptosis and improving cellular resilience postoperatively [12, 16]. These findings suggest that miRNAs could act as indicators of myocardial stress and serve as therapeutic targets to optimize cardioprotection strategies during surgery.

Importantly, when interpreted collectively, the identified miRNAs appear to converge on key pathways involved in cardioplegic ischemia–reperfusion injury, particularly apoptosis regulation, mitochondrial stability, and inflammatory signaling. For instance, suppression of miR‐15a‐5p was associated with increased Bcl‐2 expression, suggesting reduced apoptotic activation, while alterations in miR‐194‐5p levels may reflect adaptive responses to hypothermic circulatory arrest. Similarly, the release of cardio‐enriched miR‐208b and miR‐499‐5p after cardioplegia‐reperfusion likely represents myocardial cellular stress and injury signaling. These findings suggest that miRNA modulation during cardioplegic arrest may reflect both injury severity and intrinsic protective responses, although causal relationships remain to be established.

The study by Wang et al. demonstrated a notable reduction in miR‐194‐5p levels after surgery, suggesting that lower expression might contribute to reducing myocardial injury [12]. This aligns with preclinical evidence pointing to the protective role of certain miRNAs in reducing oxidative stress and inflammation during cardiac ischemia‐reperfusion injury. Additionally, studies investigating simvastatin pre‐treatment indicate that modulation of miRNA expression (e.g., miR‐15a‐5p) can enhance cardioprotection, reinforcing the potential for pharmacological interventions targeting miRNA pathways [12, 13, 14, 15].

Preclinical studies, such as those by Kiss et al., emphasize the widespread changes in miRNA profiles induced by cardioplegia and cardiopulmonary bypass [20]. These alterations were found to differ depending on the type of cardioplegia (e.g., cold *vs.* warm), with cold cardioplegia linked to a more pronounced decrease in certain cardioprotective miRNAs, such as miR‐451 [20]. These results suggest that miRNA expression is highly sensitive to surgical techniques and conditions, offering an opportunity to refine cardioplegic strategies to optimize outcomes.

Clinical studies also provide valuable evidence regarding the diagnostic and prognostic roles of miRNAs. For example, elevated levels of miR‐126 and miR‐499 were strongly correlated with postoperative outcomes, such as ejection fraction and atrial fibrillation, after coronary artery bypass grafting (CABG) [18, 21]. These findings reinforce the potential of miRNAs not only as therapeutic agents but also as robust biomarkers for predicting surgical outcomes and tailoring perioperative management.

The molecular mechanisms underlying miRNA‐mediated cardioprotection appear to involve the modulation of apoptosis, oxidative stress, and inflammation. For instance, miR‐21, miR‐1, and miR‐133 have been shown to regulate pathways involved in cardiomyocyte survival and repair [17].

The upregulation of anti‐apoptotic miRNAs like miR‐21 during ischemia‐reperfusion injury underscores their therapeutic potential. Furthermore, miRNAs such as miR‐146a and miR‐145, which influence redox signaling, may provide additional layers of protection against ischemia‐induced cellular damage.

### 4.1 Limitations

This review has several limitations. Despite a comprehensive search strategy, only five studies met the inclusion criteria, reflecting the limited and emerging nature of research on microRNAs in the specific context of cardioplegia. The cumulative sample size of cardioplegia‐exposed patients was relatively small, and considerable heterogeneity existed in surgical procedures and study designs.

In addition, key intraoperative variables—such as cardioplegia type, cross‐clamp time, cardiopulmonary bypass duration, and precise timing of miRNA sampling—were inconsistently reported in the primary studies, limiting more detailed comparative analyses. These constraints arise from reporting gaps in the original investigations rather than methodological shortcomings of this review. Finally, due to heterogeneity and limited randomized data, findings were synthesized narratively without meta‐analysis.

## 5 Conclusion

This review underscores the emerging significance of miRNAs in the field of cardiac surgery, particularly during cardioplegia. Although substantial progress has been made in understanding the role of miRNAs in cardiovascular diseases, their application in the context of cardioplegic myocardial protection remains largely unexplored. Current evidence highlights their dual utility as biomarkers and therapeutic targets. Upregulated miRNAs such as miR‐208b and miR‐499‐5p, as well as downregulated ones like miR‐194‐5p, have shown potential as mediators of cardioprotection during ischemia‐reperfusion injury.

In conclusion, while miRNAs are already recognized as crucial players in cardiovascular biology, their integration into clinical practice as tools for myocardial protection during cardioplegia offers an exciting frontier. Continued research in this domain could pave the way for novel therapeutic strategies, ultimately improving outcomes for patients undergoing cardiac surgery.

## Author Contributions

A.D. and P.S. were responsible for designing the study. P.S., F.M., and M.B. all contributed to conducting the study and drafting the manuscript.

## Funding

The authors have nothing to report.

## Conflicts of Interest

The authors declare no conflicts of interest.

## Transparency Statement

The Pourya Shokri affirms that this manuscript is an honest, accurate, and transparent account of the study being reported; that no important aspects of the study have been omitted; and that any discrepancies from the study as planned (and, if relevant, registered) have been explained.

## Data Availability

The data that support the findings of this study are available from the corresponding author upon reasonable request.
